# Multifocal lesions of the lungs, skin, bones, and brain

**DOI:** 10.1016/j.jdcr.2023.08.021

**Published:** 2023-09-12

**Authors:** Eloy E. Ordaya, Daniel M. Ries, Nikifor K. Konstantinov

**Affiliations:** a Division of Public Health, Infectious Diseases, and Occupational Medicine, Mayo Clinic, Rochester, Minnesota; b Department of Internal Medicine, Regions Hospital, St Paul, Minnesota; c Department of Dermatology, University of New Mexico School of Medicine, Albuquerque, Minnesota

**Keywords:** blastomycosis, disseminated blastomycosis, endemic fungal infection, PET/CT

## History

**Fig 1:**
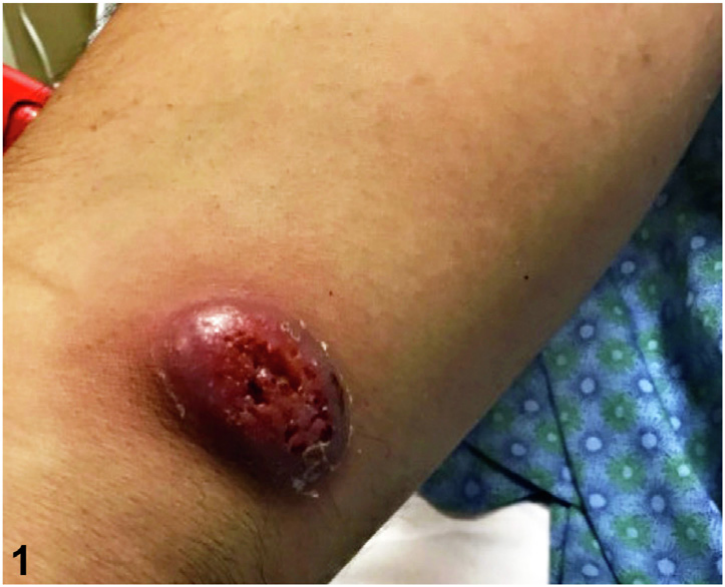

**Fig 2:**
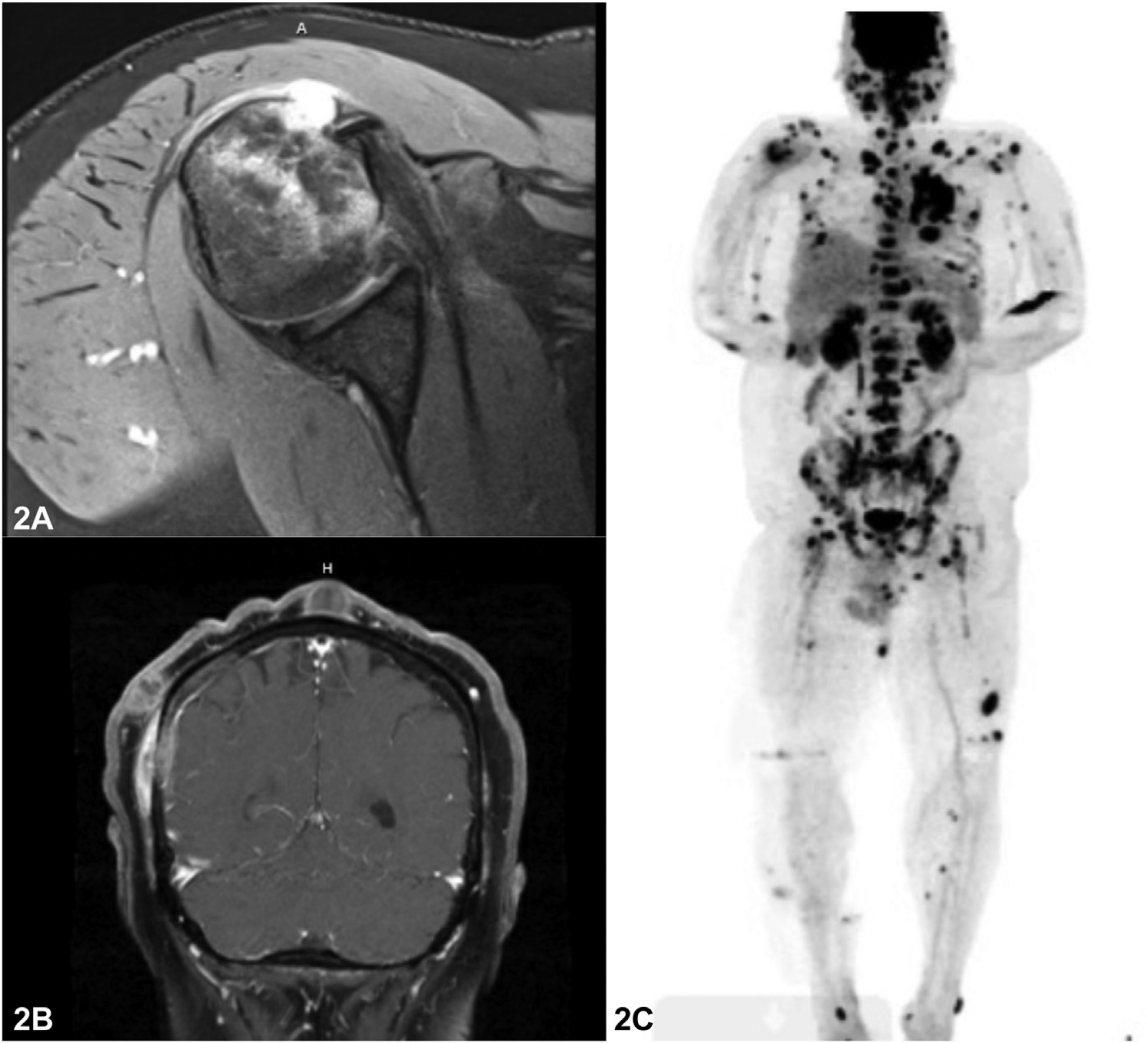

**Fig 3:**
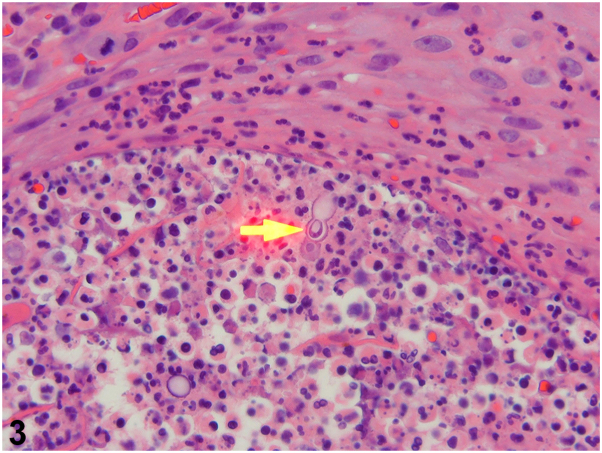

A previously healthy 34-year-old male from Minnesota presented with acute right hip and shoulder pain. He had a 5-month history of scattered violaceous cutaneous nodules involving his face, trunk, and extremities (Fig 1), accompanied by cough and headaches. HIV testing was negative. Chest X-ray revealed a left upper lobe lung infiltrate. Magnetic resonance imaging of the right shoulder showed cortical bone destruction (Fig 2, *A*). Brain magnetic resonance imaging demonstrated pachymeningeal enhancement (Fig 2, *B*), and positron-emission tomography/computed tomography (PET/CT) (Fig 2, *C*) used to determine the extent of disease revealed involvement of subcutaneous tissues, lungs, and bones (Fig 2, *B*). Punch biopsies of a nodule for hematoxylin and eosin (Fig 3) and cultures were performed.

**Question 1: What is the most likely diagnosis?**

A. Eruptive keratoacanthomas
B. Primary cutaneous cluster of differentiation (CD)8^+^ aggressive epidermotropic cytotoxic T-cell lymphoma
C. Disseminated histoplasmosis
D. Disseminated coccidioidomycosis
E. Disseminated blastomycosis

**Answers:**

A. Eruptive keratoacanthomas – Incorrect. Histopathology of keratoacanthomas would show a crateriform tumor with both endophytic and exophytic components, a large central keratin plug, and marked squamous epithelial proliferation.
B. Primary cutaneous CD8^+^ aggressive epidermotropic cytotoxic T-cell lymphoma – Incorrect. Although patients may present with disseminated eruptive ulcerated nodules, with a propensity to disseminate to visceral sites (lungs, testes, central nervous system [CNS], and mucosa), histopathology would show an epidermotropic atypical lymphoid infiltrate with markers showing expression for CD3, CD8, CD7, protein tyrosine phosphatase receptor type C, T-cell beta chain antigen receptor, and T-cell intracytoplasmic antigen, not seen in our case.^1^
C. Disseminated histoplasmosis – Incorrect. Although clinical presentation and geographic distribution of *Histoplasma capsulatum* is similar to *Blastomyces dermatitidis*, the histopathology does not fit. Histoplasmosis appears as small budding yeasts clustered within histiocytes.^2^
D. Disseminated coccidioidomycosis – Incorrect. The organism appears as doubly refractile spherules 10 to 100 um in diameter containing multiple endospores. Coccidioidomycosis is also seen most commonly in the southwestern United States.^2^
E. Disseminated blastomycosis – Correct. Blastomycosis is a fungal infection caused by the dimorphic fungi *Blastomyces* species. *B dermatitidis* and *Blastomyces gilchristii* are commonly found in the soil of various midwestern and eastern states in North America. Other *Blastomyces* species are found in Africa and the Middle East.^3^ Pulmonary infection is the most common presentation, followed by skin involvement. Cutaneous findings are characterized by verrucous papules and nodules, ulcerative plaques, or subcutaneous lesions. Disseminated disease is frequently seen in immunocompromised hosts. However, it may also occur in immunocompetent patients. This patient also had positive *Blastomyces* urine antigen and *B dermatitidis* grew in tissue culture.

**Question 2: What is the gold standard diagnostic modality for disseminated blastomycosis?**

A. Polymerase chain reaction
B. Tissue culture
C. PET/CT
D. Urine and/or blood antigen testing
E. Serologic detection

**Answers:**

A. Polymerase chain reaction – Incorrect. Polymerase chain reaction is promising but not widely available for clinical practice for blastomycoses. There is cross-reactivity with other endemic fungi, so this may be a useful diagnostic modality to decrease false positives seen in other diagnostic modalities, such as antigen testing.^4^
B. Tissue culture – Correct. Growth in culture is the gold standard for diagnosis. *Blastomycosis* species growth on culture can take up to 4 weeks. Culture samples can be taken from respiratory specimens (sputum or bronchoalveolar lavage), tissue biopsy, or cerebrospinal fluid.^4^ Histopathology or direct microscopy from sterile source showing characteristic fungal elements are also used as criteria for proven endemic mycosis according to the 2021 guidelines from the European Organization for Research and Treatment of Cancer and the Mycoses Study Group Education and Research Consortium.
C. PET/CT – Incorrect. PET/CT scanning is occasionally used as an alternative diagnostic modality for invasive fungal infections. Lesions of blastomycosis show high fluorodeoxyglucose avidity on PET/CT scanning, which may mimic a primary or metastatic malignant disease. There could be a more expanded use of PET/CT scanning in finding occult sites of infection and as a tool in monitoring treatment response, though it is not standard of care yet.^5^
D. Urine and/or blood antigen testing – Incorrect. Antigen testing is a reported sensitivity between 76% and 93% in patients with blastomycosis. Antigen testing may be performed on urine, serum, bronchoalveolar lavage, or cerebrospinal fluid. There is cross-reactivity with other endemic fungi (histoplasmosis, paracoccidioidomycosis, and talaromycosis).^4^
E. Serologic detection – Incorrect. Serology has variable sensitivity and specificity. A newer method of enzyme immunoassay to detect antibodies against the blastomyces adhesin 1 protein has both higher sensitivity and specificity but not commercially available yet.^3^

**Question 3: What is the most appropriate therapy in this patient?**

A. Intravenous (IV) amphotericin for 4 weeks
B. IV amphotericin for 2 weeks followed by an azole for 6 to 12 months
C. IV amphotericin for 4 weeks followed by azole for 1 year
D. Azole for 12 months
E. Azole for 6 months

**Answers:**

A. IV amphotericin for 4 weeks – Incorrect. Patients with severe pulmonary blastomycosis, severe extrapulmonary disease, or CNS blastomycosis, require induction therapy with amphotericin B followed by oral therapy for 6 to 12 months.^3^
B. IV amphotericin for 2 weeks followed by an azole for 6 to 12 months – Incorrect. This is the treatment regimen for severe pulmonary blastomycosis or disseminated blastomycosis without CNS involvement.^3^
C. IV amphotericin for 4 weeks followed by azole for 12 months – Correct.Patients with CNS disease, as our patient, should be treated with a lipid formulation of amphotericin B for 4 to 6 weeks followed by an azole for at least 12 months and until CNS abnormalities have resolved.^3^ Our patient received 6 weeks of amphotericin B followed by itraconazole for 1 year. At 6 months follow-up visit, his skin lesions completely resolved, chest X-ray demonstrated resolution of the lung infiltrate, and magnetic resonance imaging of the pelvis showed resolution of bone lesions.
D. Azole for 12 months – Incorrect. This may be appropriate in a patient with mild to moderate pulmonary disease or mild to moderate disseminated disease; however, in a patient with CNS involvement, induction therapy is needed.^3^
E. Azole for 6 months – Incorrect. This may be an appropriate treatment regimen for a patient with mild to moderate pulmonary involvement.^3^

## Conflicts of interest

None disclosed.
